# Personalized Surgical Tactics for an Adult Patient with Mitral Insufficiency and Dextrocardia with Situs Inversus Totalis

**DOI:** 10.21470/1678-9741-2024-0234

**Published:** 2025-12-10

**Authors:** Boris N. Kozlov, Konstantin A. Petlin, Evgeniya V. Lelik, Natalya L. Afanasieva, Yulia A. Arsenyeva, Yulia N. Chernykh, Elena B. Kim

**Affiliations:** 1 Cardiovascular Surgery Department, Cardiology Research Institute, Tomsk National Research Medical Center, Russian Academy of Sciences, Russian Federation

**Keywords:** Dextrocardia, Situs Inversus Totalis, Mitral Valve Posterior Leaflet Chord Rupture, Mitral Insufficiency, Mitral Valve Repair

## Abstract

We present a clinical case of mitral insufficiency in a 59-year-old patient with dextrocardia and complete transposition of the viscera. The patient underwent mitral valve posterior leaflet repair and annuloplasty. During the operation, a “mirror inversion” of the equipment and surgery team position was carried out. The special feature of the operation was due to the fact that the aorta and great vessels in the wound were mirror-image. The postoperative period proceeded without complications. Being aware of the patient’s dextrocardia and hence organizing the surgical procedure appropriately, we could achieve good results in radical surgery for valvular heart disease.

## INTRODUCTION

**Table:** 

Abbreviations, Acronyms & Symbols	
CPB	= Cardiopulmonary bypass
ECG	= Electrocardiogram
MSCT	= Multislice computed tomography

Dextrocardia is a rare condition that occurs in 1/8,000 to 1/25,000 newborns, its incidence among both sexes is approximately the same. Among all congenital cardiovascular defects, dextrocardia accounts for no more than 3%^[1]^.

There are scarce data on cardiac surgery for dextrocardia^[2-6]^. In this report, we present a rare clinical case of surgical treatment of a patient, who had a congenital anomaly of dextrocardia and a complete transposition of the viscera, with mitral insufficiency caused by the ruptured chord of the posterior mitral leaflet. Such heart anatomy contributes to certain challenges in a traditional setup, which include performing usual surgical “right-hander’s” procedures with the left hand, adding complexity for a surgeon to approach the mitral valve. In these settings, the good exposure of the mitral valve is practically unfeasible. Therefore, the surgery requires special considerations, such as rearranging the surgical team and equipment in a "mirror-image" setup, including the longer cardiopulmonary bypass (CPB) lines, due to the reversed anatomical orientation. All these listed factors increase the risk of errors associated with the human factor and related to the activities of the surgeon, the assistant, and the operating nurse. Therefore, we present our positive experience with operating room and equipment transformation, which can be reproduced in other clinics.

## CASE PRESENTATION

A 59-year-old male patient was electively admitted to the Cardiac Surgery Department of the Cardiology Research Institute in January 2024, presenting with complaints of dyspnea during walking.

Complete transposition of the viscera was detected in this patient at the age of 12 during a medical examination at school. He had no complaints at that time, was actively involved in sports, and did not seek medical assistance. Notably, the patient was the first-born of twins; however, the second twin died at birth due to an undetermined cause. Upon analyzing the family history, the patient did not recall any obvious congenital defects among his immediate relatives. Furthermore, his two children also do not have any congenital anomalies.

Upon admission to the Cardiac Surgery Department, the physical examination revealed notable findings: the apical impulse was palpable on the right at the midclavicular line; the borders of relative cardiac dullness were displaced, with the left border along the left edge of the sternum, the upper border in the third intercostal space to the right of the sternum, and the right border along the right midclavicular line in the fifth intercostal space. Additionally, the liver edge was palpable in the left hypochondrium.

**Fig. 1:**
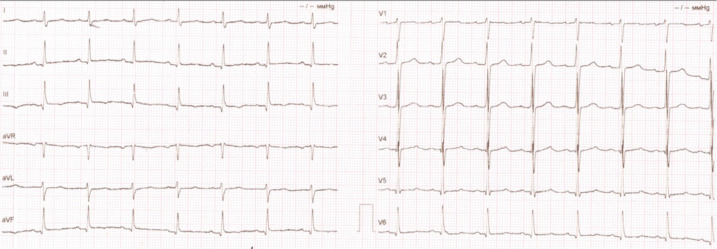
Electrocardiogram records before surgery.

Taking dextrocardia into account while recording electrocardiogram (ECG), the principles of electrode placement were deliberately changed, namely: the red electrode was placed on the left hand, the yellow one on the right hand. The chest leads were placed sequentially in a mirror-image position on the right side: V3R, V4R, V5R, V6R, V1, and V2 were swapped. ECG showed sinus rhythm with a heart rate of 90 bpm, normal electrical cardiac axis, and transition zone at V2-V3 (Figure 1).

The chest x-ray showed a complete transposition of the viscera. The pulmonary pattern was deformed due to hilar fibrosis without focal infiltrative changes.

**Fig. 2:**
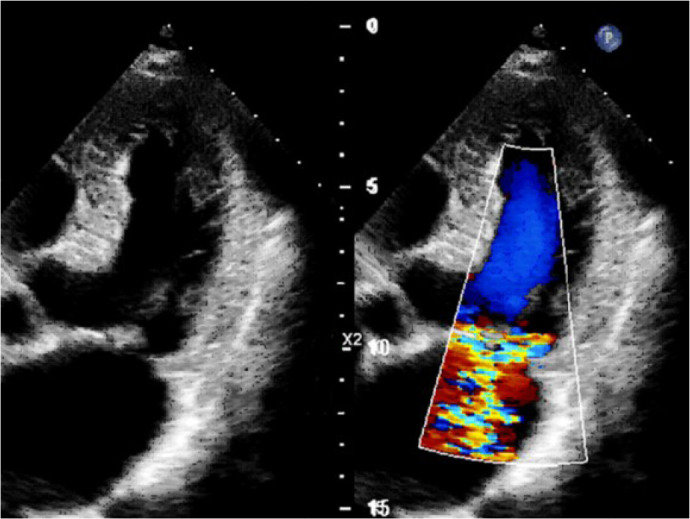
Transthoracic Doppler echocardiography demonstrating severe mitral regurgitation.

Transthoracic echocardiography (Figure 2) showed the mirror-imaged arrangement of the studied organs in comparison with the typical ultrasound image, *i.e.*, the left-developed right-sided heart. A slight left atrial enlargement was detected (52*57 mm in the four-chamber view) without chamber hypertrophy, with normal left ventricular contractility (left ventricular ejection fraction in B-mode was 66%). A slightly dilated mitral annulus (36 mm) was detected; the ruptured chord of the posterior mitral leaflet in P3 segment and grade 2 mitral regurgitation were visualized. The effective regurgitant orifice was 21 mm. Other valves were functioning normally. The pericardium was not changed. The performed carotid Doppler sonography showed that the carotid artery wall was thickened; heterogeneous plaques of up to 10% were detected in carotid bifurcation, and the internal carotid artery orifices were on both sides.

### Surgical Technique

The patient underwent invasive coronary angiography showing no signs of coronary atherosclerosis.

Thus, based on the preoperative examination, a myxomatous degeneration of the mitral valve was diagnosed with the ruptured chord of the posterior mitral leaflet in P3 segment and severe mitral regurgitation against the background of complete transposition of the viscera.

**Fig. 3:**
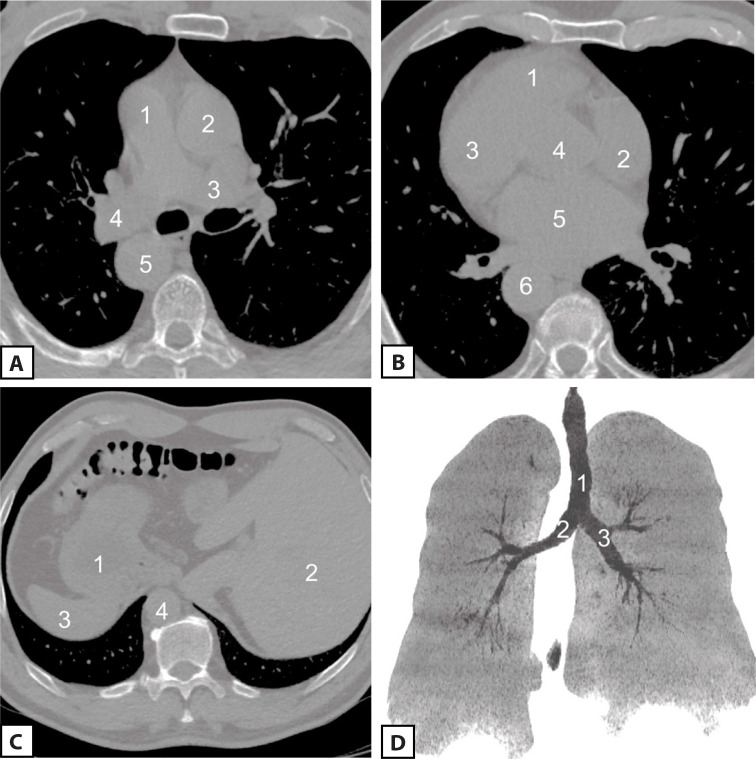
Preoperative multislice computed tomography (MSCT). A) Chest MSCT scan at the level of great vessels (1, pulmonary artery trunk; 2, ascending aorta; 3, left pulmonary artery; 4, right pulmonary artery; 5, descending thoracic aorta); B) chest MSCT scan at the level of heart chambers (1, right ventricle; 2, right atrium; 3, left ventricle; 4, an outlet of the left ventricle; 5, left atrium; 6, descending thoracic aorta); C) upper-abdominal MSCT scan (1, stomach; 2, liver; 3, spleen; 4, descending thoracic aorta); D) lung MSCT scan (1, trachea; 2, left lung developed on the right side; 3, right lung developed on the left side).

When setting out for the upcoming surgery, it was considered reasonable to perform an additional multislice computed tomography (Figures 3 A, B, C, and D).

**Fig. 4:**
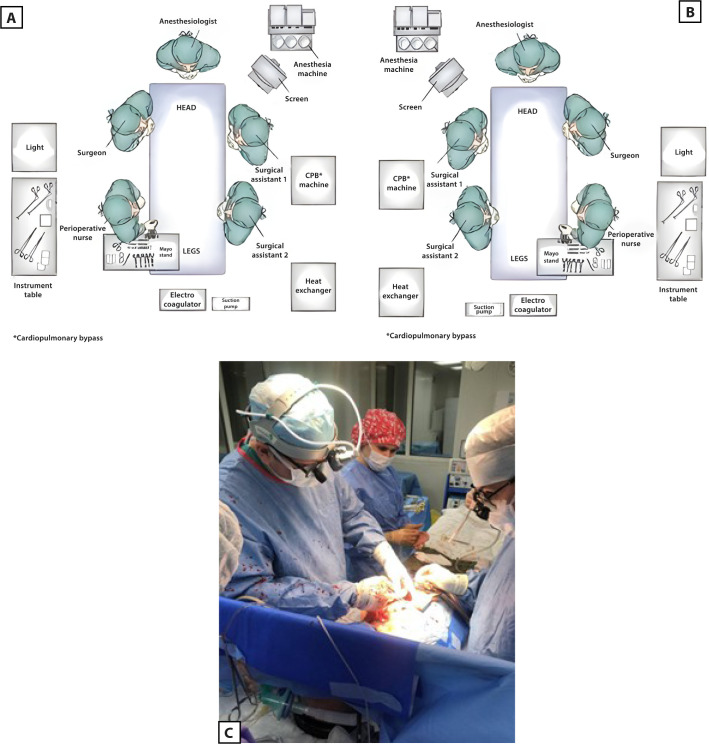
The surgical team and equipment arrangement in the operating room. A) A pictorial diagram of the surgical team and equipment position for the patient with a normal heart location; B) a pictorial diagram of the surgical team and equipment position for the patient with dextrocardia; C) photograph taken during the surgical procedure. CPB=cardiopulmonary bypass.

Due to dextrocardia and complete transposition of the viscera in the patient, certain organizational measures were to be taken. During the operation, a “mirror inversion” of the equipment (CPB machine, operating table, screens) and the position of the surgical team (operating surgeon, assistants, perioperative nurse) was carried out in the operating room (Figures 4 A, B, and C).

**Fig. 5:**
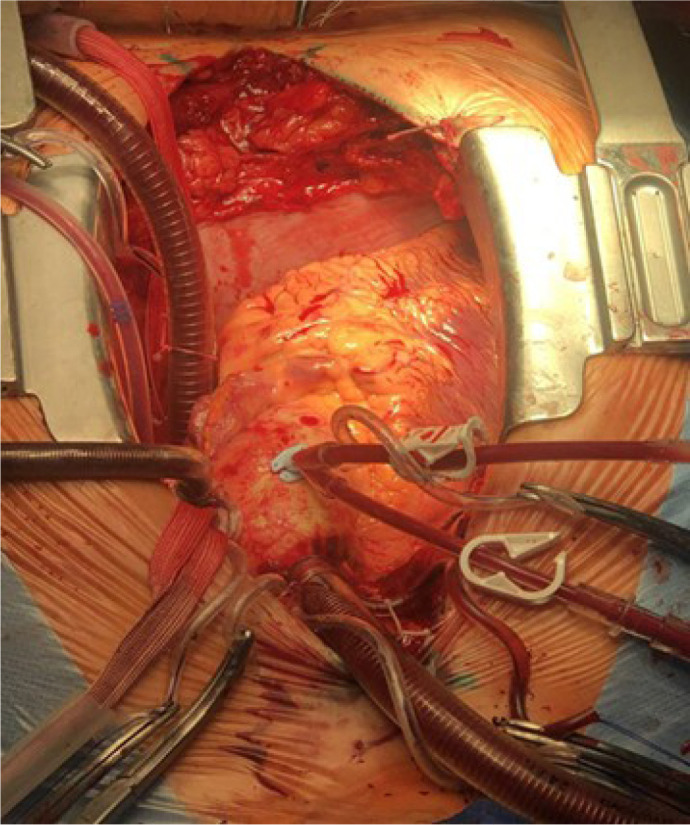
An intraoperative photograph shows a still frame of dextrocardia with visible right atrial venous cannulation on the left side of torso and adjacent distal ascending aortic cannulation.

The patient underwent posterior mitral valve leaflet repair and mitral valve annuloplasty under CPB and antegrade cold cardioplegia. The surgical access was gained typically via median sternotomy. The special technical feature was that the aorta and great vessels in the wound were mirrored from the normal position. Purse string sutures for cannulation of the aorta and vena cava were technically placed in a standard manner, but the placement itself was in the left parts of the surgical wound, *i.e.*, “non-standard” (Figure 5).

Access to the mitral valve was obtained through atriotomy performed on the left flank of the surgical wound. After the mitral valve revision and identification of changes (the fibrous ring was not dilated, the posterior leaflet of the mitral valve was thickened, the ruptured chord was at P3 segment), posterior mitral valve leaflet repair was performed. A support C-Ring was inserted in mitral position. An intraoperative transesophageal echocardiography confirmed that mitral regurgitation was not observed.

The postoperative period was uneventful. On day 10 after surgery, the patient was discharged from the hospital in a satisfactory condition.

## DISCUSSION

The abnormal right-sided location of the heart in the chest was first described by the Italian anatomist and surgeon Hieronymus Fabricius, in 1606. There are dextrocardia with situs viscerum inversus totalis - the complete reversal of internal organs (observed in our patient) - and isolated dextrocardia characterized by a right thoracic heart with normal locations of the stomach, liver, and spleen^[2-4]^. If the normal blood flow in the vessels and chambers of the heart is maintained, then this cardiac anomaly does not require any treatment. This is confirmed by the case we describe. The patient, up to 57 years of age, led an active sports lifestyle. However, the very fact of identifying dextrocardia in a patient should be an alert for cardiologists in terms of possible concomitant defects in the development of the heart, systemic dysplasias, which require dynamic monitoring and regular assessment of intracardiac structures and possible cardiac complications. For example, myxomatous degeneration of the mitral valve leaflets, which is described herein, was caused by a congenital defect of connective tissue^[5]^. The surgical technique for a patient with dextrocardia is not distinctive, but cardiac surgeons may encounter technical issues due to the mirror-image transposition of the internal organs and intracardiac structures. According to Rammos K et al.^[7]^, complications can arise with CPB connecting because the vena cava and right atrium are located more posteriorly than normal, and surgeons may be confused by mirror-image findings. Some authors describing cardiac surgery in dextrocardia and transposition of the internal organs note the particular nature of surgical interventions in such patients, especially in such emergency cases as acute aortic dissection^[6-9,10]^. Additionally, there may be a problem when performing usual surgical “right-hander’s” procedures with the left hand. Some authors recommend that the surgeon stand to the left of the patient, which provides excellent exposure because the patient’s anatomy is mirror transformed^[11,12]^.

Dealing with this rare condition, it is very important for the surgeon to have spatial abilities in order to better plan the course of the upcoming operation, to imagine three-dimensional models of the heart, internal organs, and their topography relative to each other^[6,7]^, as well as predict possible risks.

## CONCLUSION

The combination of degenerative mitral valve disease with mitral insufficiency and situs inversus in an adult patient is a rare clinical case. Full awareness of the cardiac surgery team of dextrocardia in the patient as well as the appropriate organization of the surgical procedure allowed us to achieve desired immediate results in radical surgery for valvular heart disease, which ensures clinical stability in the patient not only at the hospital stage but also in the long-term postoperative period.

Our positive experience will be useful for other clinics to apply our scheme regarding the surgical team’s new configuration in practice.

## Data Availability

The authors declare that the data supporting the findings of this study are available within the article.

## Artificial Intelligence Usage

The authors declare that no artificial intelligence tool was used in the preparation of this article.
